# 

**Published:** 1835-01-01

**Authors:** 


### CONTENTS

OF THE

### MEDICAL QUARTERLY REVIEW,

### No. VI. JANUARY 1, 1835.

---

I.

Observations on Functional Affections of the Spinal Cord and Ganglionic System of Nerves. By William Griffin, m.d., and Daniel Griffin, m.r.c.s. . . . . . . 243

II.

A Treatise on Lesser Surgery, or the Minor Surgical Operations. By Bourgery, d.m.p. Translated from the French, with Notes, and an Appendix, by W. C. Roberts, and J. B. Kissam . . 275

III.

Traité des Hemorrhagies Internes de l'Uterus, &c.

A Treatise on Internal Uterine Hemorrhage. By A. C. Baudelocque 285

IV.

Illustrations of the Elementary Forms of Disease. By Robert Carswell, m.d., Professor of Pathological Anatomy in the University of London. Fasciculus VI. Hemorrhage . . . . 298

V.

Pathological and Practical Researches on the Diseases of the Brain and the Spinal Cord. By John Abercrombie, m.d. Third Edition, enlarged 301

VI.

Clinical Lectures in the Manchester Royal Infirmary. By Edward Carbutt, m.d. . . . . . 308

VII.

The Principles of Ophthalmic Surgery; being an Introduction to a Knowledge of the Structure, Functions, and Diseases of the Eye, embracing New Views of the Physiology of the Organs of Vision. By John Walker, Esq. . . . . . 314

VIII.

Practical Treatise on Diseases of the Eye. By Wm. Mackenzie, m.d. Second Edition . . . . . 324

IX.

Traité, Théorique et Pratique, des Blessures par Armes de Guerre; rédigé d'après les Leçons Cliniques de M. le Baron Dupuytren; et publié sous sa direction par MM. les Docteurs A. Paillard et Marx. Tome Second . . . . . 339

*Reviews continued.*

X.

A Systematic Treatise on Comparative Physiology, introductory to the Physiology of Man. Translated, with Notes, from the German of FREDERICK TIEDEMANN, Professor of Anatomy and Physiology in the Heidelberg University, by JAMES MANBY GULLY, M.D., and J. HUNTER LANE, M.D., F.L.S. &c. Vol. I. . . . 351

XI.

A Practical Treatise on Lepra Vulgaris; to which are added, Observations on the Treatment of some of the Local Varieties of Psoriasis. By EDWARD BECK, M.D. . . . . 371

XII.

Illustrations of the Natural History of Worcestershire. By CHARLES HASTINGS, M.D. . . . . . 376

XIII.

A Compendium of Osteology, being a Systematic Treatise on the Bones of the Human Body; designed for the Use of Students. To which is subjoined, an improved Method of Preparing Bones for Osteological Purposes. By GEORGE WITT, M.D. . . . 384

XIV.

Illustrations of the Botany and other Branches of the Natural History of the Himalayan Mountains, and of the Flora of Cashmere. By J. FORBES ROYLE, Esq., F.L.S., &c. . . . 388

XV.

The Study of Medicine. By JOHN MASON GOOD, M.D. Fourth Edition. By SAMUEL COOPER, Esq. . . . . 391

XVI.

The Elements of Anatomy. By JONES QUAIN, M.D. . . 396

XVII.

A Popular View of Homœopathy. By the Rev. THOMAS EVEREST . 399

XVIII.

Lectures on the Ordinary Agents of Life, as applicable to Therapeutics and Hygiene. By ALEXANDER KILGOUR, M.D. . . 402

XIX.

The Surgeon's Practical Guide in Dressing, and in the Methodic Application of Bandages. Illustrated by Numerous Engravings. By THOMAS CUTLER, M.D. . . . . . 405

XX.

The Medical Pocket Book, for 1835. By JOHN FOOTE, Jun.

The Medical Almanack . . . . 407

*Reviews continued.*

XXI.

An Essay on Clinical Instruction. By E. Ch. A. Louis, M.D. Translated by Peter Martin, M.R.C.S. . . . . . 408

XXII.

Outlines of a New System of Philosophy, being a View of the System of Sciential Medicine: or Medicine (and all Human Knowledge) as proveable as Geometry. By Thomas Eden, M.R.C.S. . . 410

### Original Communications.

I.

Retrospect of the Late Improvements in Medicine, Surgery, &c. By the Editor . . . . . 413

II.

On the Formation of Artificial Pupil, without injury to the Crystalline Lens or its Capsule. By Frederick Tyrrell, Esq. . . 424

III.

Cases extracted from the Note-book of Henry Davies, M.D. . 430

IV.

Remarks on Aneurisms of the Cerebral Arteries: with Cases. By Thomas King, M.R.C.S. . . . . 434

V.

Account of several Cases of Carditis: with Remarks. Communicated to the Harveian Society, by William Stroud, M.D. . . 439

VI.

A Case of Loss of Nose from Syphilis, and Restoration by the Taliacotian Operation; with Remarks, by Frederick Tyrrell, Esq. . 448

VII.

Case of Ulcerated Cæcum. By F. E. Hicks, Esq. . . 457

### COLLECTANEA.

### PATHOLOGY AND PRACTICE.

Animal Magnetism . . . . . 459

Case of Apparent Hæmoptysis caused by a Leech . . 460

Case of Strangulated Hernia followed by Cholera . . ib.

Cases of Hysterical Affection of the Knee . . . 461

Case of Adhesion of the Placenta to the Fundus of the Uterus . 463

Fungus Hæmatodes . . . . . 464

Ligature of the Aorta . . . . . ib.

On Belladonna in Pertussis . . . . ib.

Treatment of Nasal Polypi . . . . . . 465
The Itch Insect . . . . . . 466
Case of Ununited Fracture, successfully Treated by Friction . 467
Effects of the Solution of Muriate of Antimony in Carcinoma. By Dr. A. Neumann . . . . . . 469
Carbonate of Ammonia in the Urine . . . 470
Account of a Trial of Acupuncture with Galvanism, made by Dr. W. Stokes, one of the Physicians to the Meath Hospital. By John Hamilton, L.R.C.S.I. . . . . 472
Capivi in Catarrh of the Bladder, and Leucorrhœa . . 475
On the Diagnosis of Fractures of the Neck of the Femur . 477
Instance of Destruction of the Uterus, Perineum, and Rectum, after Delivery, with Recovery . . . . 480
Case of Varicose Veins . . . . 481
Difficulty of Diagnosis . . . . . 482
Case of Hepatic Abscess . . . . 483
Notes of a Case of Fistulous Opening of the Stomach, successfully treated by Dr. J. H. Cook . . . . . 484
Use of Opium in Mania . . . . 485

### MISCELLANEOUS.

Charitable Establishments at Florence . . . 486
Cause of the Colours of Plants . . . . 488
Experiments on Atomic Weights. By Dr. Turner . . 489
Vegetation near Quito . . . . . 490
The Platysma Myoides . . . . 491
Mortality from Phthisis at Different Ages . . . 492
Excretions of Plants . . . . . ib.
Entries in the Old Bills of Mortality . . . 493
Falsification of Writings . . . . 494
Accidental Vaccination . . . . ib.
The Death of Hannibal . . . . . 495

### INTELLIGENCE.

The Royal Medical and Chirurgical Society . . . 496
Meteorological Register. Notices . . . . 498

